# Mapping the gap: Misalignment between emergency care research and consensus priorities in the Western Cape

**DOI:** 10.4102/jcmsa.v3i1.226

**Published:** 2025-07-29

**Authors:** Robert Holliman, Colleen J. Saunders

**Affiliations:** 1 Division of Emergency Medicine, Department of Family, Community and Emergency Care, Faculty of Health Sciences, University of Cape Town, Cape Town, South Africa

**Keywords:** evidence mapping, research priorities, Western Cape, low- and middle-income countries, knowledge translation

## Abstract

**Background:**

The Western Cape (WC) province of South Africa is one of the highest emergency care (EC) research-producing regions in Africa. In 2021, a consensus exercise with key stakeholders established 26 EC research priorities. This study aimed to confirm evidence gaps in relation to the priorities and assess the alignment between frontline knowledge needs and research output within the WC EC community.

**Methods:**

We developed an evidence map of all EC research published from the WC between January 2017 and December 2021 to describe the alignment of each publication with any previously established priority. Additional data were extracted from all studies that addressed one or more of these priorities.

**Results:**

A total of 246 EC publications were identified, with 24% (*n* = 60) addressing one or more of the 26 established priorities, while five priorities remained unaddressed. Priority-aligned papers appeared in 36 journals, of which 16% (*n* = 41), including seven priority-aligned papers, were behind a pay wall. Most priority-aligned studies were observational (48%) or qualitative (23%), with only two systematic reviews and no experimental studies.

**Conclusion:**

Less than a quarter of recent EC research publications from the WC addressed established consensus priorities, confirming the existence of consensus evidence gaps and suggesting potential misalignment between research output and community-identified needs.

**Contribution:**

This study provides an evidence-based assessment of how well EC research in the WC reflects community-established priorities. The findings highlight the need for stronger alignment between research production and frontline knowledge needs to maximise impact and reduce research waste.

## Introduction

It is a well-established fact that delivering effective emergency care (EC) has the potential to dramatically improve the health of people living in low- and middle-income countries (LMIC), with the World Bank Disease Control Priorities Project estimating that over half of LMIC deaths can be attributed to conditions potentially manageable with emergency treatment.^1^ High-quality EC systems are reliant on robust research that targets evidence gaps to establish treatment standards and improve practice.^2^ However, this evidence should be both implementable and context specific, with standards of treatment derived from research applicable to local populations. Clinicians in LMICs face a significant challenge as most EC research is still conducted in high-income countries (HICs), much of which investigates the use of advanced resources and systems often unavailable in LMICs. In addition, research findings from these settings are often not generalisable to LMIC populations because of pathophysiologic differences, variations in treatment response and disparities in baseline risk, all of which can affect intervention efficacy and efficiency.^3^ Replication studies that implement practices derived from HIC research have not consistently demonstrated the same improved outcomes in LMICs and, in some cases, have indicated potential harm.^4,5^ Contextually appropriate research, involving local populations within the local context and health system, is therefore essential to ensure appropriate EC can be delivered.

Multiple barriers to research production in LMICs have been identified, including difficulty accessing published work,^6,7^ difficulty attending medical conferences and meetings^8^ and a lack of funding, training and time.^9^ This is particularly evident in Africa, with under 1% of global EC literature addressing practice on the continent.^10^ Strategies such as establishing regional research priorities can improve the impact and efficiency of studies conducted and prevent wasted effort.^11^ The process of establishing research priorities should be inclusive, transparent and specific enough to develop appropriate questions, ensuring that the limited resources available address areas with the most potential for impact.^12^

In 2021, Holliman et al. conducted a modified consensus study to establish EC research priority needs for the Western Cape (WC) province,^13^ a region that contributed approximately 60% of EC publications from South Africa, and 20% of those from Africa, between 2017 and 2021.^14,15^ The study identified 26 specific research questions across four key areas: pre-hospital on-scene care, pre-hospital transport, facility-based EC and EC systems. Although consensus studies remain contentious because of potential biases that may influence outcomes,^16^ these techniques provide a structured approach to priority setting and, by involving key local stakeholders, the identified priorities are likely to be relevant to the local patient population and clinicians. However, consensus methods alone cannot fully determine the extent of existing research, the magnitude of knowledge gaps or the need for further investigation.

Mapping is an evidence synthesis method used primarily as a tool to help identify knowledge gaps within specified research areas and set agendas for future work. As noted by Miake-Lye et al., the technique has yet to undergo the same level of scrutiny and development as other methodologies such as rapid or scoping reviews, and a standardised process for mapping has not yet been established.^17^ However, they identify five common components of evidence mapping: (1) identifying evidence gaps or needs; (2) providing user-friendly outputs; (3) addressing a broad field; (4) following a systematic process; and (5) using visual depictions of results.^17^ James et al. describe evidence mapping as most appropriate for characterising the current state of knowledge on a topic rather than synthesising the results to answer a specific question.^18^ It is therefore useful when a review is likely to collate highly heterogeneous studies that make systematic synthesis challenging. Mapping can help explore aspects such as the volume of existing evidence, the populations, interventions, exposures or outcomes studied and the methodologies employed in these studies.^18^

In its most basic form, evidence mapping can identify research topics with a low volume of outputs or those investigated using a limited number of study designs. By mapping the recent EC research output from the WC against the established consensus priorities for the community, we can determine the volume and type of research conducted that attempts to address these research questions. Mapping can, therefore, provide additional evidence that specific topics are, in fact, priorities and provide an indicator of the level of alignment between research output and research needs for a region. A higher volume of good-quality studies focusing on subjects deemed to be priorities for a region could be considered a marker of alignment between those producing research and those predominantly utilising research outputs. The aim of this study was, therefore, to construct an evidence map of EC research outputs from the WC for the five years preceding a previous study that established consensus research priorities for the region (January 2017–December 2021) and cross-index these publications against the established priorities to assess the degree of alignment between the reported research needs and actual research gaps.

## Research methods and design

This observational study builds on the initial work conducted by Holliman et al. and represents the second phase of a broader project, which began with a 2021 consensus exercise that identified 26 EC priorities for the WC.^13^ We employed an evidence mapping approach to identify all EC research published within the academic and research-producing community of the WC in the five years prior to the 2021 priority-setting exercise. As a recently established form of evidence reviewing, we drew on the work of James et al.^18^ and Miake-Lye et al.^17^ to design the study and include the major components of an evidence mapping study.

Initially, we contacted the primary EC research-producing higher education institutions (HEIs) within the WC and requested a list of emergency medicine research outputs between January 2017 and December 2021. We then conducted a supplementary online search using the SciVal (Elsevier, Amsterdam, Netherlands) database to identify all research publications classified under the subject area ‘Emergency Medicine’ from HEIs within the WC. The lead author (R.H.) screened all articles against the inclusion and exclusion criteria outlined in Table 1. All eligible articles were then retrieved and downloaded for analysis.

**TABLE 1:** Inclusion and exclusion criteria for publication analysis.

Inclusion criteria	Exclusion criteria
Studies registered or published involving at least one author who was affiliated with an HEI within the WC at the time of publication Studies related to the subject area of Emergency medicine Studies published between January 2017 and December 2021	Research focused on practice or systems within the WC but does not involve at least one author affiliated with an academic institution within the WC Research where the primary focus is not emergency care related

HEI, higher education institutions; WC, Western Cape.

The lead author (R.H.) initially evaluated the titles, abstracts and keywords of all identified articles to ascertain whether the study aligned with the research priorities established in the previous consensus study. Three additional reviewers then independently conducted a second review of the same fields to confirm alignment. If there was any uncertainty as to whether a publication addressed a given priority, the full text was retrieved and reviewed. To ensure consistency in interpretation, all reviewers participated in an initial pilot review of a sample of articles. During this phase, reviewers independently assessed a subset of publications and then discussed their findings collectively to resolve discrepancies and refine the approach. This process helped maintain inter-rater reliability and develop a shared understanding of how alignment with priorities would be judged. Discrepancies and queries were resolved through consensus between the authors (R.H., C.J.S.).

Articles identified as addressing at least one of the agreed research priorities then underwent further data abstraction or coding.^18^ Information including the study design, year of publication, publication journal, study setting and whether the publication was open access was captured.

Statistical analysis of the volume and percentage of publications addressing the established priority list was then performed. All data analysis was performed using Microsoft Excel® (Microsoft Corporation, Redmond, Washington) with categorical data presented as frequencies where appropriate.

### Ethical considerations

Ethical clearance to conduct this study was obtained from the University of Cape Town Faculty of Health Sciences Human Research Ethics Committee (No. HREC 299/2023).

## Results

**FIGURE 1:**
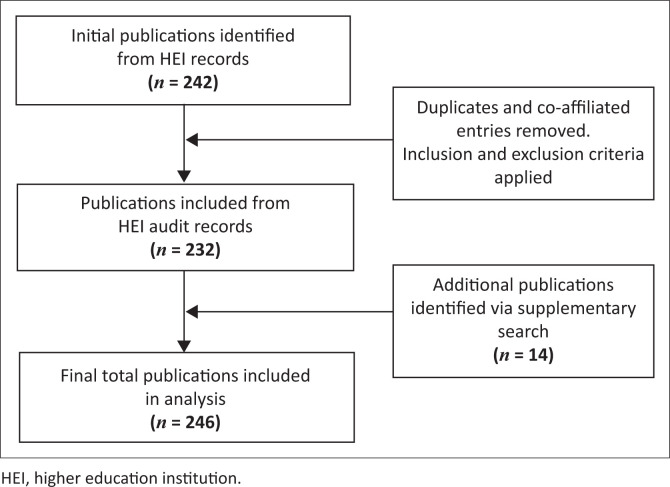
Screening and selection process for included publications.

Following the initial search, it was determined that a total of four WC HEIs produced EC research between January 2017 and December 2021. These included the University of Cape Town (UCT), Stellenbosch University (SU), Cape Peninsula University of Technology (CPUT) and the University of the Western Cape (UWC). A total of 151 publications were retrieved from UCT, 82 from SU, 5 from CPUT and 4 from UWC. There was considerable overlap in authorship, with many researchers holding co-affiliations across multiple HEIs, particularly between UCT and SU. After the removal of duplicate records and repeat entries arising from co-affiliated authors and shared outputs across institutions, and following the application of inclusion and exclusion criteria, the final dataset consisted of 232 EC research articles produced between January 2017 and December 2021. In the supplementary search, we identified an additional 14 publications that involved at least one author from an HEI within the WC, bringing the total number of publications to 246. A flow diagram illustrating the screening and selection process is presented in Figure 1.

**FIGURE 2:**
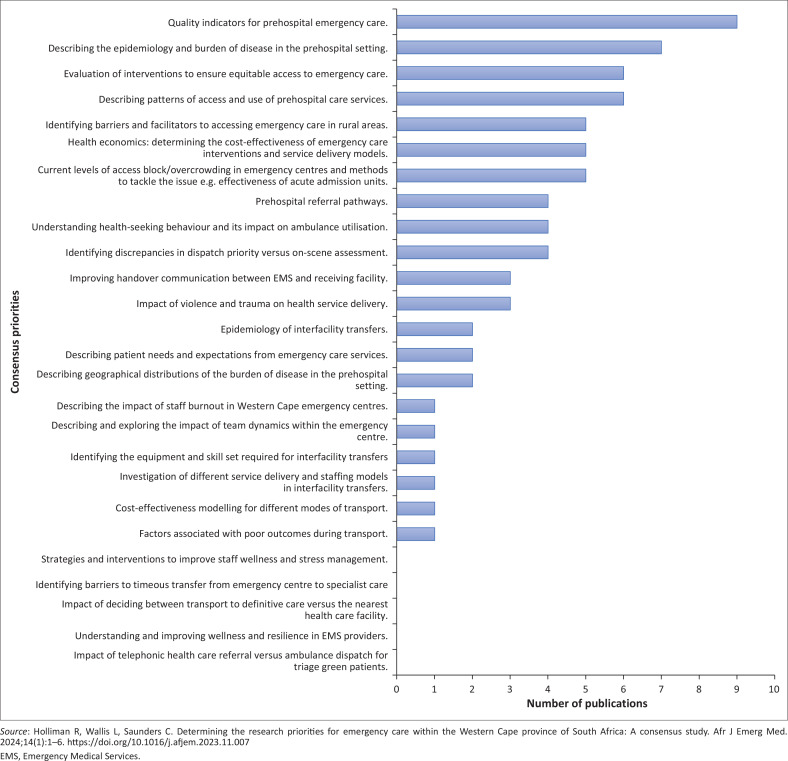
Number of publications per previously identified research priority.

In total, 24% (*n* = 60) of these articles focused at least partially on one or more of the previously established consensus priorities, which means that 76% (*n* = 186) of the published articles did not address an established priority for the region. Out of the 26 consensus priorities identified previously, 80% (*n* = 21) were partially addressed by at least one article, while five priorities remained wholly unaddressed (Table 2^13^ and Figure 2^13^).

**TABLE 2:** Volume of publications between January 2017 and December 2021 addressing research priorities established by Holliman et al.

Emergency care research priority	Addressed by at least one publication	Number of publications addressing priority
**Pre-hospital on-scene EC**		
Impact of telephonic health care referral versus ambulance dispatch for triage green patients.	No	0
Identifying discrepancies in dispatch priority versus on-scene assessment.	Yes	4
Understanding health-seeking behaviour and its impact on ambulance utilisation.	Yes	4
Describing patterns of access and use of pre-hospital care services.	Yes	6
Understanding and improving wellness and resilience in EMS providers.	No	0
Describing the epidemiology and burden of disease in the pre-hospital setting.	Yes	7
Describing geographical distributions of the burden of disease in the pre-hospital setting.	Yes	2
Quality indicators for pre-hospital emergency care.	Yes	9
Impact of violence and trauma on health service delivery.	Yes	3
Describing patient needs and expectations from emergency care services.	Yes	2
**Pre-hospital transport**		
Factors associated with poor outcomes during transport.	Yes	1
Pre-hospital referral pathways.	Yes	4
Cost-effectiveness modelling for different modes of transport.	Yes	1
Improving handover communication between EMS and receiving facility.	Yes	3
Impact of deciding between transport to definitive care versus the nearest health care facility.	No	0
Epidemiology of interfacility transfers.	Yes	2
Investigation of different service delivery and staffing models in interfacility transfers.	Yes	1
Identifying the equipment and skill set required for interfacility transfers.	Yes	1
**Emergency care facilities**		
Identifying barriers to timeous transfer from emergency centre to specialist care.	No	0
Current levels of access block and overcrowding in emergency centres, and methods to tackle the issues	Yes	5
Describing and exploring the impact of team dynamics within the emergency centre.	Yes	1
Strategies and interventions to improve staff wellness and stress management.	No	0
Describing the impact of staff burnout in Western Cape emergency centres.	Yes	1
**Emergency care systems**		
Health economics: determining the cost-effectiveness of emergency care interventions and service delivery models.	Yes	5
Identifying barriers and facilitators to accessing emergency care in rural areas.	Yes	5
Evaluation of interventions to ensure equitable access to emergency care.	Yes	6

*Source:* Holliman R, Wallis L, Saunders C. Determining the research priorities for emergency care within the Western Cape province of South Africa: A consensus study. Afr J Emerg Med. 2024;14(1):1–6. https://doi.org/10.1016/j.afjem.2023.11.007

EMS, Emergency Medical Services; EC, emergency care.

A quarter of all articles (*n* = 62, 25%) were published in the *African Journal of Emergency Medicine* (AfJEM), including 20% (12 of the 60) of the priority-aligned papers. The *South African Medical Journal* (SAMJ) and the *South African Journal of Pre-Hospital Emergency Care* (SAJPEC) contributed 13 and 6 of the total publications, respectively. The 60 priority-aligned papers were distributed across 36 different journals, reflecting a broad dissemination of research outputs. Of the 246 total publications, 16% (*n* = 41) were published behind a paywall. This includes 11.6% (7 of the 60) priority-aligned publications restricting their availability to those without institutional access or subscriptions. From the 60 publications addressing at least one priority, 48% (*n* = 29) were observational, normally involving retrospective analysis, or qualitative in design, 23% (*n* = 14). There were no identified randomised controlled trials and only two systematic reviews focusing on any of the set priorities (Table 3).

**TABLE 3:** Extracted data from the 60 priority-aligned publications.

Data	Research publications	
	*n*	%
**Study Design**		
Observational	48.3	29
Qualitative	23.3	14
Reviews (systematic and scoping)	10.0	6
Mixed and quasi methodology	8.3	5
Consensus and commentary	6.6	4
Other	3.3	2
Experimental	0.0	0
**Journal**		
International	61.6	37
Regional (African)	26.6	16
Local (South African)	11.6	7
**Setting**		
Pre-hospital	42.0	25
Emergency care facilities	30.0	18
Other	2.0	12
Hospital and Pre-hospital	5.0	3
Interfacility transfer	3.0	2
**Open Access**		
Yes	86.6	52
No	13.3	8

## Discussion

Through this basic evidence mapping review we were able to describe the EC research outputs from the WC and assess their alignment to consensus-established priorities from the same region. With only 24% of all WC EC research addressing identified priorities and no publications focusing, either directly or indirectly, on five of the 26 research questions, the identified evidence gaps were confirmed.

The significant lack of recent research focusing on topics agreed to be priority areas by key stakeholders potentially indicates a misalignment between research output and information needs with a large volume of publications exploring subjects and questions that were not identified as priorities for the region. Concerns regarding misalignment of clinical research and public health needs are not a new concept and have been highlighted previously with several studies demonstrating the poor correlation between public funding and disease burden,^19,20^ as well as the mismatch between patient-desired management strategies and the interventions actually studied.^21^ In a previous mapping exercise reviewing 115 000 randomised controlled trial (RCTs) conducted globally, Ata et al.^22^ identified significant research gaps in all regions outside of HICs, with sub-Saharan Africa being particularly affected. Their analysis highlighted a stark mismatch between research focus and disease burden, revealing that only 6% of RCTs investigated common infectious diseases and just 2% addressed neonatal disorders, despite these conditions accounting for 23% and 12% of the region’s disease burden, respectively.^22^ Mismatches in research publications and priorities are likely multi-factorial with proposed factors including commercial and donor funding bias, vested researcher interests and a lack of end-user involvement in research.^21^ Additionally, factors related to research publication may further exacerbate this misalignment, with publication bias, particularly the preferential reporting of statistically significantly positive results, skewing the available evidence base.^23^ While not all research must align strictly with established priorities – there remains an important role for investigating under-prioritised health concerns, emerging issues and novel ideas – there should nonetheless be a concerted effort to ensure that a meaningful proportion of research addresses agreed-upon regional needs.

An additional consideration is the overall value and effectiveness of consensus-driven priority lists in shaping research agendas. A previous study conducted in the WC found that only 7% of EC research publications from two major HEIs in the region aligned with a set of national research priorities established in 2015 through a consensus process.^24,25^ While this finding is in alignment with the results of our study, it also raises questions about the long-term impact of static, once-off priority-setting exercises. Consensus processes provide a structured approach to identifying research needs, but without sustained engagement between researchers and the broader clinical community, their influence on actual research output may be limited. A more dynamic, iterative approach to priority setting, integrated with ongoing dialogue between research producers, clinicians and policymakers, may better ensure that emerging challenges are identified and investigated in a timely and impactful manner. The majority of publications addressing priorities were observational or qualitative in study design (43% combined). Because of the diverse range across the 26 priorities, determining the most suitable methodology to effectively address these research questions is challenging. It is also uncertain whether the relatively homogeneous nature of study design is less ideal than a more heterogeneous spread. Nevertheless, these research designs often occupy lower positions on established evidence hierarchy charts or pyramids and are therefore commonly accepted as relatively lower levels of evidence.^26^ Of note there were only two systematic reviews, generally seen as one of the highest forms of evidence, that focused on priority questions. Our sample of EC research generated within the WC aligns with prior studies examining outputs from LMICs highlighting a lower citation impact and generally lower level of evidence, especially from the African region.^27^

Publication patterns and accessibility play a critical role in determining the reach and impact of EC research. In our study, AfJEM emerged as the most prominent publication venue, accounting for a quarter of all publications, including the highest number of priority-aligned papers. As the most widely recognised and highest-indexed EC-specific journal based in Africa, AfJEM provides a key regional platform for disseminating research relevant to the WC. The presence of SAMJ and SAJPEC as other notable journals reflects the role of locally based journals in supporting research with direct implications for the South African healthcare system. Of note, the latter journal was discontinued in 2023 and is no longer accepting submissions or publishing new content. This not only raises uncertainty about the future visibility and discoverability of its previously published articles but also represents the potential loss of a valuable publishing platform for regional EC research. Despite the role of key regional journals, WC EC research outputs, particularly those aligned with established priorities, were published across a wide range of journals. While diverse publication avenues may enhance global visibility, many international journals are behind international currency paywalls, which can limit access for policymakers and clinicians in resource-limited settings. Ensuring open-access publication in such journals is therefore critical to maximise the reach and utility of priority-aligned research. A substantial proportion (13.3%) of publications were published behind paywalls, including several of the priority-aligned papers, limiting their availability to researchers, clinicians and decision-makers without institutional subscriptions. Our findings are consistent with previous studies examining article accessibility, including Al Hamzy et al.^6^, who found that 22% of the 500 most-cited emergency medicine articles published between 2012 and 2016 were only accessible by subscription.^6^ In LMIC settings, where financial and institutional barriers to accessing paywalled research are significant, restricted access further exacerbates challenges in evidence translation. This issue may be further compounded by the potential for poor conversion of higher degree-related research into published outputs. A previous study examining research from WC HEIs found that only 42% of successfully graded EC dissertations and theses between 2015 and 2020 had been published in a medical journal at the time of the analysis.^24^ This low publication rate creates additional barriers to knowledge translation, making it harder for stakeholders to access and apply relevant findings. Without a coordinated and meaningful approach to dissemination of research findings to stakeholders, the impact of research aligned with established priorities may be diminished. Addressing these issues is essential for ensuring that EC research informs healthcare improvements in the region. Non-priority-aligned research, ineffective methodologies and barriers to dissemination and access are significant contributors to research waste, a problem estimated to account for up to 85% of research investment being lost.^28,29^ This issue is particularly concerning in LMICs, where limited funding, shortages of skilled researchers and restricted access to scientific journals already hinder the production of high-quality evidence. In such resource-constrained settings, allocating scarce resources to research that fails to address pressing healthcare needs or lacks methodological rigour is not just inefficient but represents an ethical concern. Strengthening research governance, aligning research agendas with clinical priorities and improving dissemination are essential steps in reducing this avoidable loss.

Our study is not without limitations. As we only compiled and mapped information from the subset of publications addressing previously established research priorities, this study does not offer a comprehensive evidence map for all EC research within the WC and may not fully represent the outputs of the WC. The determination of whether a publication addressed one or more of the priorities was based on analysis of the title, abstract and, in case of doubt, the full publication, thus introducing a potential risk of subjectivity. However, the fact that all papers underwent multiple analyses by at least two separate authors should mitigate this risk. As we mapped the WC EC outputs against the results of a previous consensus exercise, any limitations or potential bias of that exercise can affect the findings of our study. In particular, the consensus priorities used for mapping reflect the period 2017–2021 and may not fully capture more recent shifts in EC knowledge needs. Additionally, the time lag between the completion of higher-degree research and formal journal publication may result in recent, relevant outputs not yet being reflected in this study. Finally, although supplementary searches were performed, the primary source for publication identification was self-audited reports on EC research outputs from WC HEIs. There is a possibility that a small number of additional publications may have been missed and not incorporated into our study.

## Conclusion

Through this mapping exercise, we established the fact that a significantly low proportion of EC research publications from the WC region between 2017 and 2021 focused, either directly or indirectly, on any of the research questions established as priorities during a recent priority-setting exercise. Most priority-aligned studies were observational or qualitative, indicating a predominance of lower levels of evidence. Research dissemination was highly fragmented across multiple journals, and accessibility remained a concern, with many publications behind paywalls. This study confirms that the topics established in previous consensus work are priorities requiring increased focus while also highlighting the need for greater alignment between research agendas and clinical priorities, improved stakeholder engagement and better strategies for ensuring research accessibility and impact.
